# Effects of strabismus surgery on choroidal blood flow: a systematic review and meta-analysis

**DOI:** 10.1186/s40942-026-00818-1

**Published:** 2026-02-23

**Authors:** Sahel Khazaei, Zahra Moravej, Mehrdad Motamed Shariati

**Affiliations:** 1 Eye Research Center, Mashhad University of Medical Sciences, Mashhad, Iran; 2 Eye Research Center, Khatam Al-Anbia Eye Hospital, Gharani Boulevard, Mashhad, Iran

**Keywords:** Strabismus surgery, Choroidal thickness, Choroidal vascularity index, Optical coherence tomography angiography, Choriocapillaris perfusion, Meta-analysis

## Abstract

**Background:**

Strabismus surgery may affect choroidal circulation, with potential implications for outer retinal nourishment and visual function. We conducted a systematic review and meta-analysis to quantify postoperative changes in subfoveal choroidal thickness and vascularity parameters following strabismus surgery.

**Methods:**

A systematic search was conducted in PubMed, Scopus, and Web of Science. After two-stage title/abstract and full-text screening, eligible observational studies reporting pre- and postoperative optical coherence tomography (OCT) or OCT angiography (OCTA) choroidal measures were included. Random-effects meta-analyses estimated pooled mean differences for subfoveal choroidal thickness (SFCT), choroidal vascularity index (CVI), foveal choriocapillaris vessel density (VD), and choriocapillaris flow. Subgroup analyses, meta-regression, quality assessment using the Risk Of Bias In Non-randomized Studies of Interventions (ROBINS-I) tool, and evaluation of publication bias were performed.

**Results:**

Eighteen studies met the inclusion criteria. Pooled analysis of 22 groups (14 studies) showed no significant overall change in SFCT after strabismus surgery (mean difference − 1.13 μm; 95% CI − 6.93 to 4.67; *p* = 0.70; I^2^ = 52.1%). CVI (seven groups, five studies) showed no significant change (pooled mean change 0.001; 95% CI − 0.006 to 0.008; *p* = 0.70; I^2^ = 0.04%). Foveal choriocapillaris VD and flow showed no significant pooled changes, though choriocapillaris VD exhibited substantial heterogeneity (I^2^ = 87.9%). Meta-regression detected no associations with follow-up duration, surgical procedure, or number of muscles. No clear publication bias was observed.

**Conclusion:**

Current observational evidence suggests no consistent alterations in SFCT, CVI, or choriocapillaris vascularity metrics during the intermediate postoperative period following strabismus surgeries.

**Supplementary Information:**

The online version contains supplementary material available at 10.1186/s40942-026-00818-1.

## Background

Strabismus, characterized by the misalignment of the eyes, poses a significant clinical challenge due to its prevalence and potential effects on visual development, binocular function, and quality of life [1, 2]. Surgical realignment of the extraocular muscles is the primary treatment approach when conservative measures prove inadequate. Although the mechanical correction of ocular deviation is the principal objective of strabismus surgery, increasing attention has been directed toward its potential effects on ocular circulation, particularly within the choroid, a highly vascularized tissue essential for outer retinal nourishment and metabolic homeostasis [3–6]. The integrity of choroidal blood flow is critical for maintaining retinal function. Disturbances in this circulation have been implicated in the pathogenesis and progression of ocular diseases, including diabetic retinopathy, age-related macular degeneration, and glaucoma [7]. Recent developments in imaging technology, particularly optical coherence tomography (OCT) and OCT angiography (OCTA), enable clinicians and researchers to non-invasively measure choroidal parameters such as subfoveal choroidal thickness (SFCT), choroidal vascularity index (CVI), and choriocapillaris perfusion. These parameters act as substitutes for assessing vascular responses post-surgical interventions. Existing evidence regarding the vascular effects of strabismus surgery remains heterogeneous and inconclusive. Some studies have reported temporary increases in choroidal thickness and vascular volume shortly post-surgery, indicating reactive hyperemia or vascular remodeling processes [8–10]. For instance, swept-source OCTA studies have demonstrated that horizontal rectus muscle surgeries result in transient elevations in choroidal thickness and vessel density, with more significant alterations observed after two-muscle procedures and lateral rectus surgeries in comparison to medial rectus operations [11]. Conversely, other studies have observed minimal or no significant vascular changes, or changes that do not persist beyond the acute postoperative phase [12, 13]. Additionally, variability in surgical techniques, patient demographics, imaging modalities, and follow-up durations has contributed to this heterogeneity and obscured definitive conclusions. Clarifying the vascular consequences of strabismus surgery has important clinical implications, particularly for patients with systemic or ocular vascular vulnerability. In such populations, alterations in choroidal perfusion could theoretically influence disease progression, visual outcomes, or postoperative recovery. Therefore, a clear understanding of the magnitude and time course of choroidal vascular changes after surgery is essential for optimizing surgical decision-making and postoperative monitoring. This study provides the first comprehensive quantitative synthesis of multiple choroidal vascular metrics, including SFCT, CVI, and choriocapillaris vessel density (CC VD) and flow, following strabismus surgery. By integrating OCT- and OCTA-derived outcomes across diverse surgical techniques and patient populations, this meta-analysis offers a unified assessment of posterior segment vascular safety after extraocular muscle surgery.

## Methods

### Protocol and guidelines

This systematic review and meta-analysis were conducted in accordance with the Preferred Reporting Items for Systematic Reviews and Meta-Analyses (PRISMA) 2020 guidelines [14]. The review protocol was registered in the International Prospective Register of Systematic Reviews (PROSPERO) (registration number: CRD420250654396) to ensure transparency and reduce the risk of reporting bias.

### Literature search

A comprehensive systematic search was conducted across three electronic databases: PubMed, Web of Science, and Scopus, encompassing all records from the database’s inception to May 2025. The search strategy combined keywords and Medical Subject Headings (MeSH) terms related to “strabismus,” “strabismus surgery,” “choroid,” “choroidal thickness,” “choroidal vascularity index,” “optical coherence tomography,” “OCT,” “OCT-angiography,” and “vascular parameters”. A comprehensive explanation of the search strategy and the specific keywords used is provided (Supplementary Table 1). Grey literature was searched using the ProQuest Dissertations and Theses Global databases. A manual screening of reference lists from included studies and relevant reviews was conducted to identify additional eligible articles. Although no language restrictions were applied during the initial search, only articles published in English were included in the final analysis. All duplicate records were identified and removed using EndNote software.

### Study selection

**Fig. 1:**
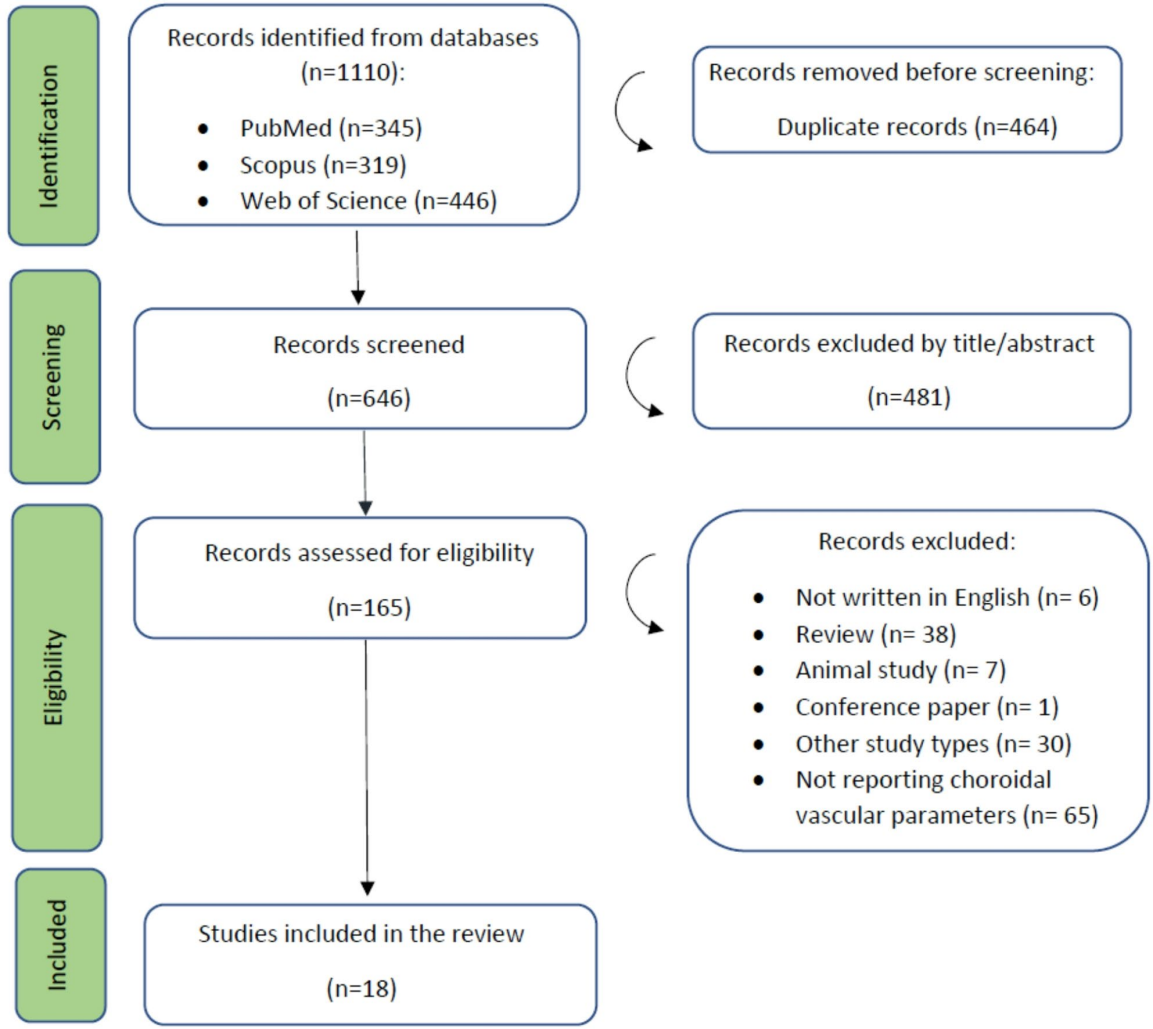
PRISMA flow diagram of the study’s selection process

Two independent reviewers (M.M. and S.Kh) screened titles and abstracts for relevance. Full texts of potentially eligible studies were retrieved and assessed against predefined inclusion and exclusion criteria. Disagreements were resolved through discussion or consultation with a third reviewer (Z.M.). The entire selection process was documented using a PRISMA 2020 flow diagram (Fig. 1).

### Eligibility criteria

Inclusion criteria were: (1) Original peer-reviewed studies published in English before May 2025 (2), human studies involving participants of any age undergoing strabismus surgery (3), studies evaluating choroidal vascularity parameters—including choroidal thickness, total choroidal area, stromal area, luminal area, CVI, or choriocapillaris vessel density—using optical coherence tomography (OCT), OCT-angiography, or similar imaging modalities (4), study designs including cohort studies and case-control studies. Exclusion criteria were: (1) studies not written in English or without an available English full text (2), studies lacking a control group or pre- and post-operative comparative data (3), studies focusing on ocular conditions other than strabismus that could influence choroidal vascularity (e.g., ocular inflammation, diabetic retinopathy) (4), reviews, systematic reviews, meta-analyses, case reports, case series, editorials, letters, conference abstracts, and commentaries (5), studies with inaccessible full texts despite attempts to contact authors (6), animal or in vitro studies.

### Data extraction

Data extraction was performed independently by two reviewers (M.M. and S.Kh) using a standardized form. The following data were extracted:

1. Study characteristics: authors, publication year, country, study design, sample size
2. Participant demographics: age, sex, type of deviation
3. Surgical details: type of surgery (e.g., recession, resection), surgical approach (fornix-based vs. limbal-based), number of operated muscles
4. Imaging modalities and parameters: OCT/OCT-angiography settings, imaging time points (pre- and post-operative)
5. Outcome measures: quantitative values of choroidal thickness, CVI, and choriocapillaris vessel density and flow pre- and post-surgery

Any discrepancies were resolved through discussion, with the assistance of a third author (Z.M.) when necessary. When data were missing or unclear, efforts were made to contact the study authors for clarification.

### Risk of bias and quality assessment

The methodological quality and risk of bias of included studies were independently assessed by two reviewers (M.M. and S.Kh) using The Risk Of Bias In Non-randomized Studies – of Interventions, Version 2 (ROBINS-I) assessment tool [15]. This tool evaluates bias across seven domains: confounding, selection of participants, classification of interventions, deviations from intended interventions, missing data, measurement of outcomes, and selection of the reported result. Discrepancies were resolved through consensus. Based on the assessment outcomes, studies were categorized as having “low,” “moderate,” “serious,” or “critical” risk of bias.

### Data synthesis and statistical analysis

Meta-analyses were conducted using a random-effects model (DerSimonian and Laird method) to account for anticipated heterogeneity between studies. For continuous outcomes (e.g., choroidal thickness, vascular parameters), we calculated mean differences (MD) with 95% confidence intervals (CIs). For continuous pre/post outcomes reported without SD of change, SD (change) was computed as sqrt (sd_pre^2 + sd_post^2 − 2*r*sd_pre*sd_post) assuming a within-subject correlation coefficient *r* = 0.5; sensitivity analyses used *r* = 0.3 and *r* = 0.7. Between-study heterogeneity was quantified using the I² statistic, with values of 25%, 50%, and 75% indicating low, moderate, and high heterogeneity, respectively. When substantial heterogeneity (I² > 50%) was detected, we explored potential sources through pre-planned subgroup and sensitivity analyses.

All statistical analyses were performed using Stata version 16 software (StataCorp, College Station, TX, USA). Publication bias was assessed visually using funnel plots and statistically with Egger’s test, when at least ten studies were included in a meta-analysis. A p-value < 0.05 was considered statistically significant. Sensitivity analyses involved excluding studies rated as having a high risk of bias to evaluate the robustness of the pooled estimates. Additional sensitivity analyses were conducted based on study design, sample size, and follow-up duration when sufficient data were available.

### Metrics and terminology

For clarity and consistency across included studies, key choroidal and choriocapillaris metrics were defined according to widely accepted OCT and OCTA conventions. Sub foveal choroidal thickness refers to the perpendicular distance between the outer border of the retinal pigment epithelium and the choroid–scleral interface measured directly beneath the fovea. The CVI is expressed as the ratio of the luminal (vascular) area to the total choroidal area within a binarized OCT image, providing a dimensionless estimate of the proportion of vascular tissue in the choroid. *Total choroidal area (TCA)*, *luminal area (LA)*, and *stromal area (SA)* represent the composite, vascular, and stromal components of the choroid, respectively. OCTA-derived *choriocapillaris vessel density* denotes the percentage of perfused vasculature within a defined region of interest in the choriocapillaris slab, while *choriocapillaris flow* indicates the quantified flow signal or flow area detected by device-specific algorithms. Because imaging platforms differ in segmentation boundaries, scan sizes, and flow-detection techniques, all metrics were extracted as reported by each study without cross-device standardization. These definitions were applied uniformly throughout the review and meta-analysis to ensure methodological coherence when synthesizing outcomes.

## Results

### Search results and baseline characteristics

The systematic search retrieved a total of 1110 records from PubMed (*N* = 345), Scopus (*N* = 319), and Web of Science (*N* = 446). After removing 464 duplicates, 646 records underwent title and abstract screening, excluding 481 for irrelevance. Full texts of 165 articles were assessed; 147 were excluded for reasons such as inappropriate study design, absence of choroidal vascular assessments, or incompatible populations. Ultimately, 18 studies met the inclusion criteria for this systematic review. The selection process for the included articles is illustrated in Fig. 1 using the PRISMA flow diagram. The search results from each database are provided in Supplementary Table 1.

**Table 1:** Characteristics of studies included in the systematic review

Author/ year	Study design	Country	Groups based on the type of surgery	*N*	Age (years), Mean ± SD	Male (%)	Follow-up duration, days	Type of OCTA/OCT	Main outcome
Xiao et al. [16], (2023)	Cross- sectional	China	One horizontal rectus muscle surgery	16	18 ± 10.96	38	7	VG100, Svision	SFCT increased more significantly after two-muscle surgery
			Two horizontal rectus muscle surgery	14	25.57 ± 9.33				
Atalay et al. [17], (2019)	Cross- sectional	Turkey	One rectus muscle surgery	26	19.76 ± 15.48	50	7	Heidelberg	Significant increase in SFCT after rectus muscle surgery; no change after IO myectomy
			IO myectomy	15	16.25 ± 10.91	46.66			
Yetkin et al. [18], (2020)	Prospective cohort	Turkey	Two horizontal rectus muscle surgery	60	9.5 ± 3.1	50	90	RTVue 100°CT	Early postoperative SFCT decreased; no significant change at later follow-up
Hashemi Javaheri et al. [19], (2024)	Prospective cohort	Iran	IO myectomy	18	24.22 ± 18.14	72.22	90	RTVue XR Avanti	Significant increase in SFCVI during first postoperative week
Alis et al. [20], (2021)	Retrospective cohort	Turkey	Horizontal rectus muscle recession	25	8.96 ± 7.96	56	180	Nidek OCT RS-3000	SFCT increased early; no difference between one- and two-muscle surgery
			Horizontal rectus muscle recession+ resection	25	15.17 ± 6.80	52			
Huseyinhan et al. [4], (2022)	Cross-sectional	Turkey	Horizontal rectus muscle surgery	33	11.18 ± 3.8	57.6	30	Topcon DRI Triton	Significant increase in CC VD one week after surgery
Meng et al. [21], (2023)	Cross-sectional	China	Two horizontal rectus muscle surgery	32	27.9 ± 11.8	40.626	7	VG100, SVision	No significant change in CC perfusion; SFCT increased with stable CVI
Uzun et al. [12], (2024)	Prospective cohort	Turkey	One horizontal rectus muscle surgery	14	9.01 ± 2.74	36	30	Zeiss Cirrus 4000	No significant postoperative SFCT change
			Two horizontal rectus muscle surgery	12	8.55 ± 2.33	58			
Yetkin et al. [22], (2023)	Prospective cohort	Turkey	IO myectomy	54	9 ± 4.6	50	90	RTVue 100 ◦CT	Early SFCT decrease, no difference after 3 months
Yetkin et al. [23], (2023)	Prospective cohort	Turkey	Horizontal rectus muscle surgery	41	12.3 ± 2.9	46.15	90	Heidelberg	Transient early postoperative SFCT decrease in standard procedure group; no significant difference in ACV conservation group
			Horizontal rectus muscle surgery + ACV conservation	38	12.6 ± 2.7	52			
Gül et al. [24], (2024)	Cross-sectional	Turkey	One horizontal rectus muscle surgery	9	23 ± 14	68	90	RTVue XR Avanti	Significant early postoperative increase in CC blood flow
			Two horizontal rectus muscle surgery	27					
			IO anteriorization	8					
Uzun et al. [9], (2022)	Prospective cohort	Turkey	One horizontal rectus muscle surgery	26	18.85 ± 11.12	38.46	30	Zeiss Cirrus 4000	Transient early SFCT decrease; no difference at 1-month post-op
Yasuda et al. [6], (2025)	Prospective cohort	Japan	All types of strabismus surgery	116	15.75 ± 3.529	53.4	120	Zeiss Cirrus 5000	SFCT increased early post-op; no significant change at 4 months
Huang et al. [11], (2025)	Prospective cohort	China	One horizontal rectus muscle surgery	59	47.45 ± 15.1	47.45	30	VG200, Svision	CCP increased with early foveal changes; CVV and CT increased at 1 week, CVI decreased, all returning to baseline at 1 month
			Two horizontal rectus muscle surgery	65	49.23 ± 11.88	49.23			
Alis et al. [25], (2024)	Cross-sectional	Turkey	Two horizontal rectus muscle surgery	42	16.7 ± 13.5	55.55	7	Nidek OCT RS-3000 Advance	Significant decrease in CVI and increase in TCA and SA on first post-op day; returned to pre-op values by day 7
Emekli et al. [26], (2024)	Prospective cohort	Turkey	Horizontal rectus muscle surgery	25	16.4 ± 6.75	45.83	30	Optovue	Significant increase in CC-flow area
Celik et al. [27], (2021)	Retrospective cohort	Turkey	IO anteriorization	72	12.26 ± 10.02	50	30	Topcon DRI Triton	Transient increase in central CC VD early post-op; no difference at 1 month
Vagge et al. [10], (2022)	Prospective cohort	Italy	At least one rectus muscle surgery	92	41 ± 22.7	54	30	Topcon DRI Triton	Transient early increase in CC VD; no difference at 1 month

Abbreviations: SFCT = subfoveal choroidal thickness; CT = choroidal thickness; CVI = choroidal vascularity index; TCA = total choroidal area; SA = stromal area; LA = luminal area; CC = choriocapillaris; VD = vessel density; IO = inferior oblique; ACV = anterior ciliary vessel; CCP = choriocapillaris perfusion; OCT = optical coherence tomography; OCTA = OCT angiography

Table 1 summarizes key characteristics of included studies conducted between 2019 and 2025 (Table 1). Geographically, 11 studies originated from Turkey, 3 from China, 1 each from Japan, Iran, and Italy. All 18 were observational in design. The parameters of the choroidal vasculature were assessed both before and after the surgery. Fourteen studies reported SFCT changes [4, 6, 9, 11–13, 18–20, 22, 23, 28–30]; five evaluated choriocapillaris vessel density and three evaluated blood flow [4, 10, 11, 13, 16, 24, 26, 27]; five investigated CVI [11, 13, 16, 19, 25]. Surgical interventions predominantly involved rectus muscle surgeries (recession, resection, or combined) in 14 studies [4, 7–9, 13, 14, 16–22]; six studies focused on inferior oblique muscle surgery [6, 19, 22, 24, 27, 29]. Only one study included all types of strabismus surgery, including operations on the rectus muscles as well as the inferior and superior oblique muscles [6]. Five studies exclusively enrolled pediatric participants [7, 9, 15, 16, 21], while others included adults or mixed populations. Regarding the surgical approach, four studies used the fornix-based method in rectus muscle surgeries [10, 21–23], five used limbal-based [4, 5, 7, 12, 16], and the remaining did not specify. Only one study assessed anterior ciliary vessel preservation techniques [23]. Follow-up periods ranged from 1 day to 6 months postoperatively, with eleven studies focusing on short-term outcomes (≤ 1 month) [4, 9–13, 16, 17, 25–27], and seven extending beyond 1 month [6, 19, 20, 22–24, 31]. Three studies employed DRI OCT Triton from Topcon Corporation using Topcon IMAGEnet 6 software [4, 10, 27]. Two studies utilized the Optovue RTVue XR Avanti (Optovue Inc., Fremont, CA, USA) with AngioVue software [24, 26], and two others used VG100 (SVision Imaging, Ltd., Luoyang, China) with its built-in software [13, 16], while one study employed VG200 (SVision Imaging Technology Co, Ltd) using built-in software version 1.28.6 [11]. These differences highlight variability in equipment and analytical methods.

### Subfoveal choroidal thickness

**Fig. 2:**
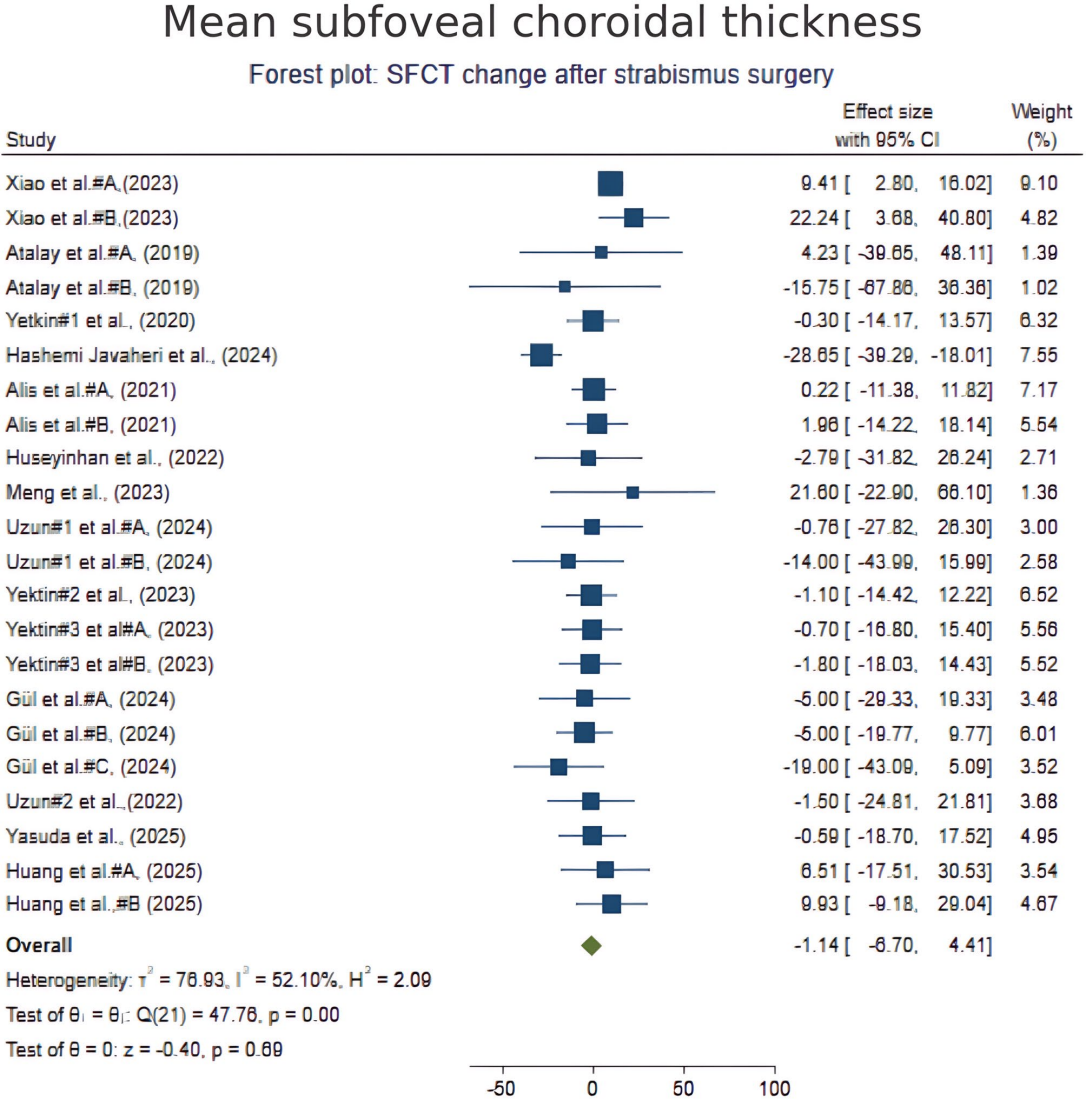
Forest plot of SFCT change after strabismus surgery

A total of 22 groups from 14 studies reporting postoperative changes in SFCT were included. Pooled analysis using a random-effects model demonstrated no significant overall change in SFCT following strabismus surgery (mean difference − 1.13 μm; 95% CI, − 6.93 to 4.67; *p* = 0.70; Fig. 2). Moderate heterogeneity was observed (I² = 52.1%). Subgroup analyses revealed that fornix-based approaches were associated with a significant reduction in SFCT (–12.3 μm; 95% CI, − 23.7 to − 0.9; *p* = 0.03), while limbal-based approaches and horizontal rectus muscle surgeries showed no significant effect. Inferior oblique procedures suggested a trend toward choroidal thinning, though it was not statistically significant. Meta-regression analysis revealed no significant associations with follow-up duration, surgical procedure, or the number of muscles operated. Funnel plot inspection and Egger’s test (*p* = 0.91) did not indicate publication bias.

### Choroidal vascular index

**Fig. 3:**
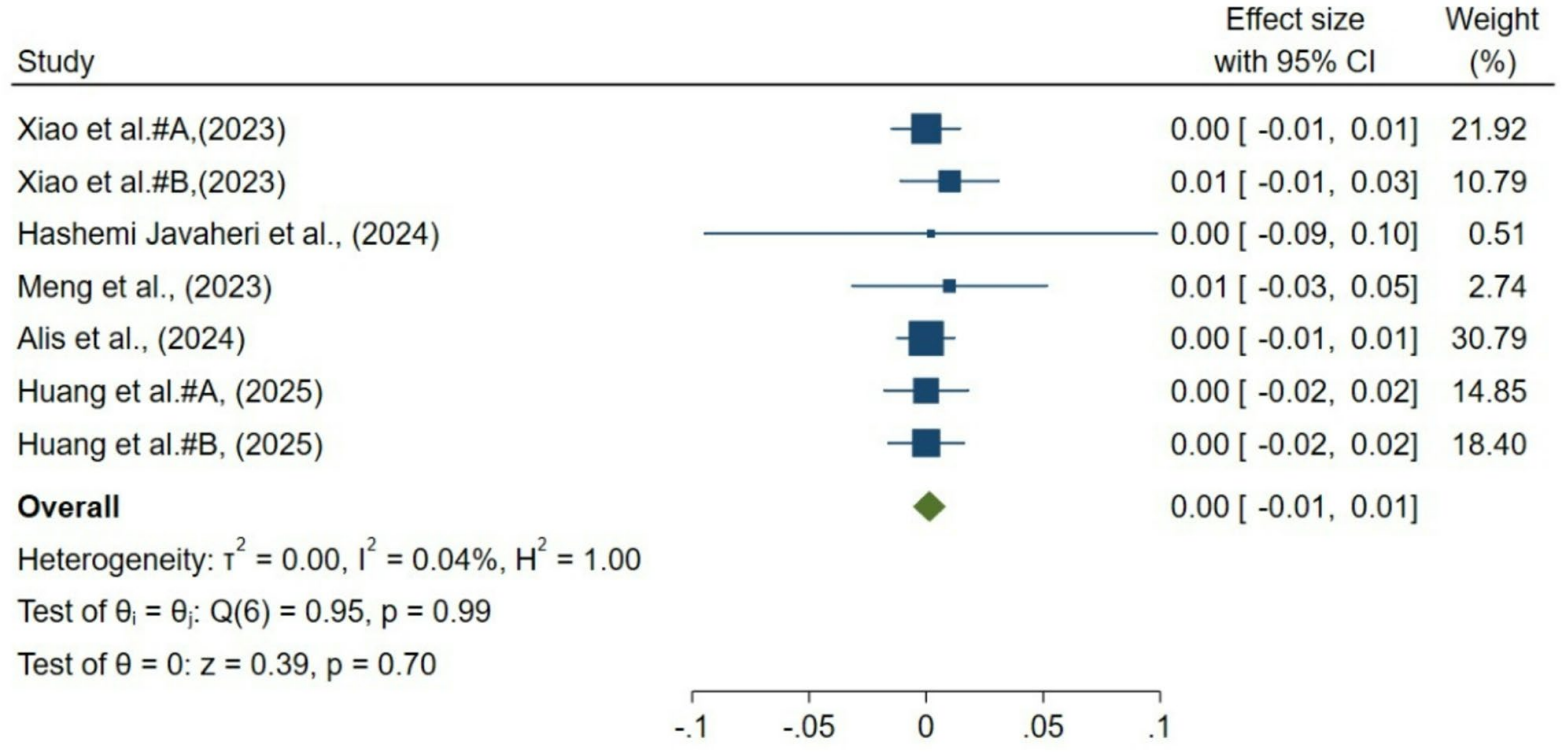
Forest plot of choroidal vascular index (CVI) change after strabismus surgery

Seven groups from 5 studies evaluated postoperative changes in CVI following strabismus surgery. Random-effects meta-analysis demonstrated no significant overall alteration in CVI (pooled mean change = 0.001; 95% CI, − 0.006 to 0.008; *p* = 0.70; Fig. 3). Heterogeneity across studies was negligible (I² = 0.04%, τ² = 0.000, Q = 0.95, *p* = 0.99), indicating consistent findings across different populations and surgical approaches. Sensitivity analyses confirmed the robustness of the results, as exclusion of individual studies did not materially affect the overall effect estimate. Publication bias was unlikely: visual inspection of the funnel plot revealed symmetry, and trim-and-fill analysis did not impute additional studies.

### Choriocapillaris vessel density

**Fig. 4:**
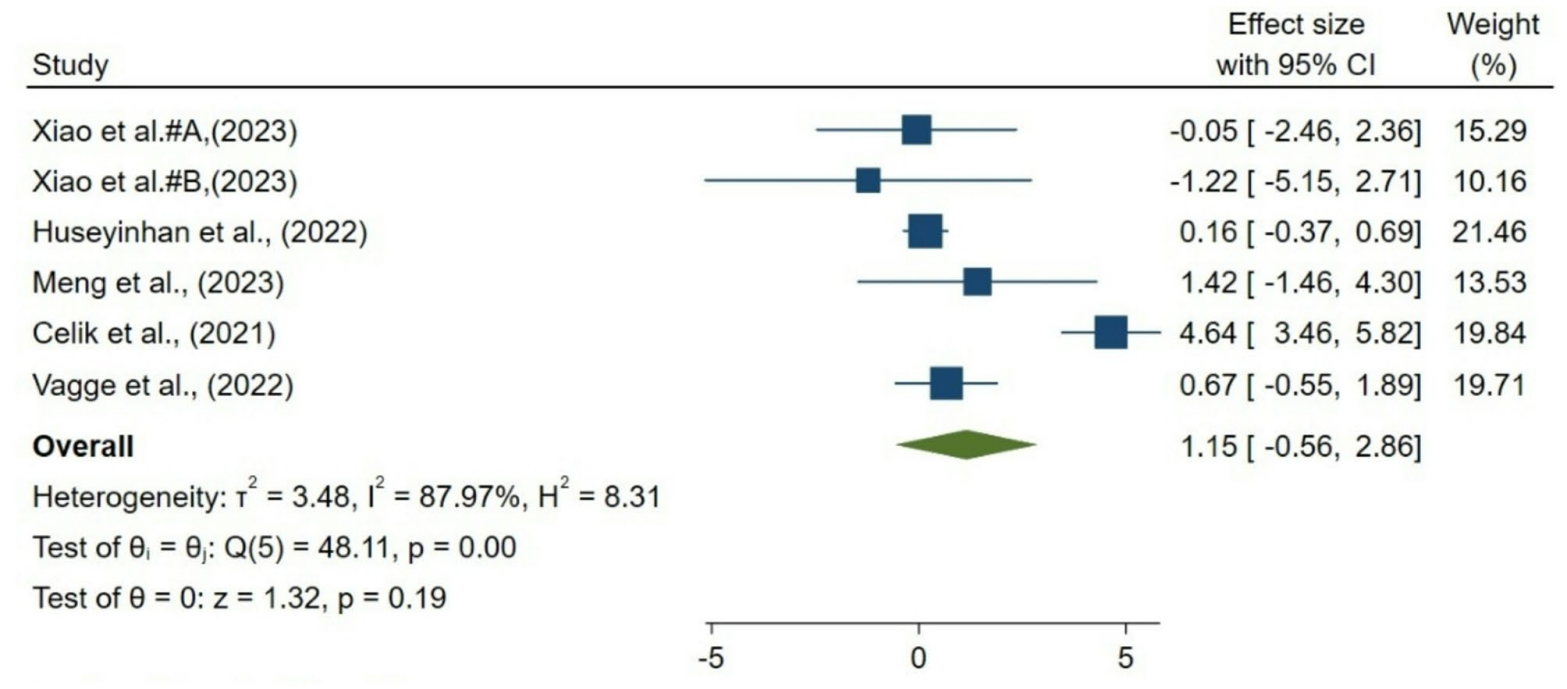
Forest plot of choriocapillaris vessel density (VD) change after strabismus surgery

Six groups from 5 studies assessed postoperative changes in foveal CC VD. The pooled random-effects analysis showed no statistically significant change in VD% following strabismus surgery (mean difference = 1.15%; 95% CI, − 0.56 to 2.86; *p* = 0.19; Fig. 4). However, substantial heterogeneity was present across studies (I² = 87.9%, τ² = 3.48, Q = 48.11, *p* < 0.001). Effect sizes varied markedly, ranging from significant increases in some cohorts (e.g., Celik et al. [27], + 4.64%, 95% CI 3.46–5.82) to null or negative changes in others. These findings indicate inconsistency in reported VD% responses, warranting cautious interpretation and exploration of methodological and procedural sources of heterogeneity.

### Choriocapillaris flow

**Fig. 5:**
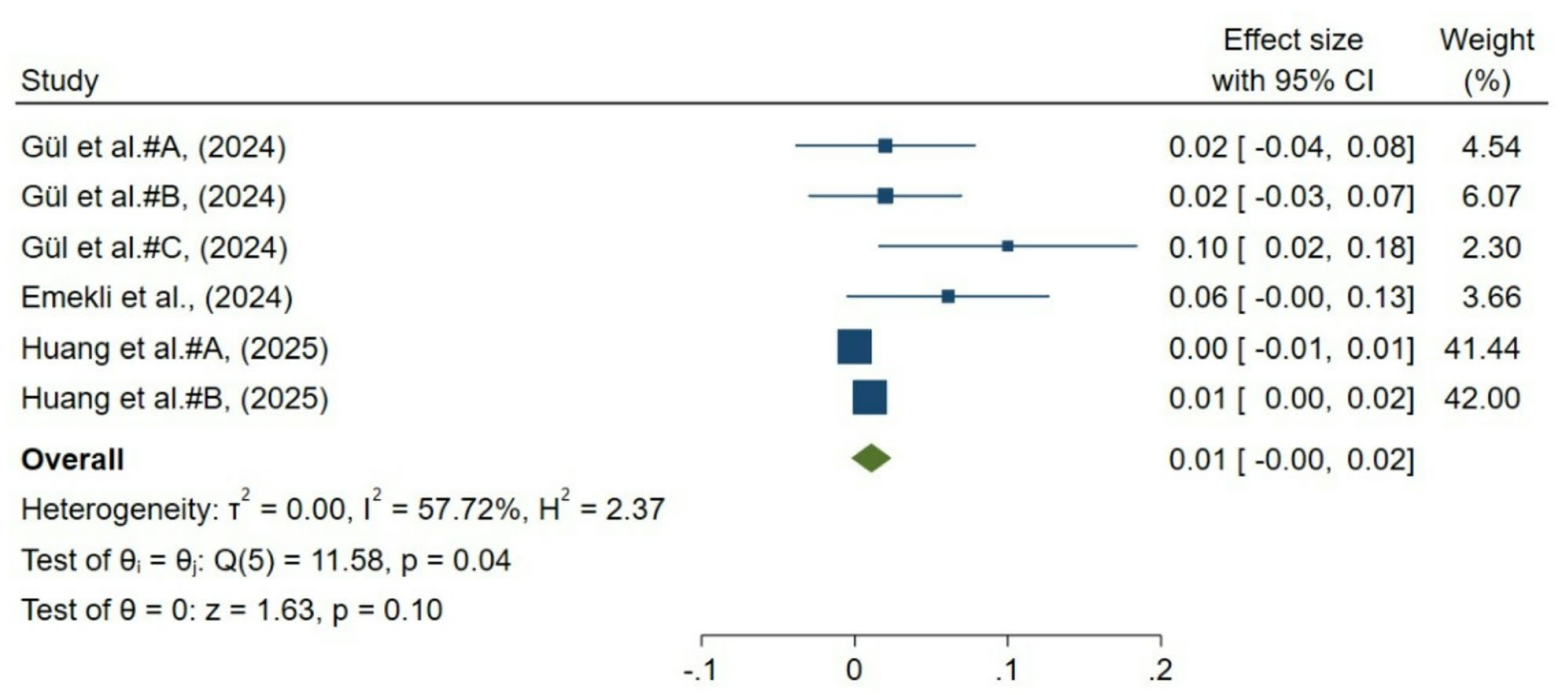
Forest plot of choriocapillaris flow change after strabismus surgery

A total of 6 groups from 3 studies reported postoperative changes in choriocapillaris flow. Pooled analysis using a random-effects model demonstrated no significant change in choriocapillaris flow (mean difference 0.01; 95% CI, − 0.00 to 0.02; *p* = 0.10; Fig. 5). Moderate heterogeneity was observed (I² = 57.72%) across studies.

### Quality of the studies

Most studies scored low risk of bias across assessed domains, with the predominant concern being uncontrolled confounding factors related to nonrandomized designs. Table 2 illustrates the quality of risk details based on their confounding, selection of participants, classification of interventions, deviations from intended interventions, missing data, measurement of outcomes, selection of the reported result, and the overall risk of bias.

**Table 2:** Details of risk of bias assessment of included studies

Authors	Confounding	Selection of participants	Classification of interventions	Deviations from intended interventions	Missing data	Measurement of outcomes	Selection of the reported result	Overall risk of bias
Xiao et al. [16], (2023)	Low (except for concerns about uncontrolled confounding)	low risk of bias	low risk of bias	moderate risk of bias	low risk of bias	low risk of bias	low risk of bias	moderate risk of bias
Atalay et al. [17], (2019)	Low (except for concerns about uncontrolled confounding)	low risk of bias	low risk of bias	low risk of bias	low risk of bias	low risk of bias	low risk of bias	low risk of bias
Yetkin et al. [18], (2020)	low (except for concerns about uncontrolled confounding)	low risk of bias	low risk of bias	low risk of bias	low risk of bias	low risk of bias	low risk of bias	low risk of bias
Hashemi Javaheri et al. [19], (2024)	Low (except for concerns about uncontrolled confounding)	low risk of bias	low risk of bias	low risk of bias	low risk of bias	low risk of bias	low risk of bias	low risk of bias
Alis et al. [32], (2021)	Low (except for concerns about uncontrolled confounding)	low risk of bias	low risk of bias	low risk of bias	low risk of bias	low risk of bias	low risk of bias	low risk of bias
Huseyinhan et al. [4], (2022)	Low (except for concerns about uncontrolled confounding)	low risk of bias	low risk of bias	low risk of bias	low risk of bias	low risk of bias	low risk of bias	low risk of bias
Meng et al. [21], (2023)	Low (except for concerns about uncontrolled confounding)	low risk of bias	low risk of bias	low risk of bias	low risk of bias	low risk of bias	low risk of bias	low risk of bias
Uzun et al. [12], (2024)	Low (except for concerns about uncontrolled confounding)	low risk of bias	low risk of bias	low risk of bias	low risk of bias	low risk of bias	low risk of bias	low risk of bias
Yetkin et al. [22], (2023)	Low (except for concerns about uncontrolled confounding)	low risk of bias	low risk of bias	low risk of bias	low risk of bias	low risk of bias	low risk of bias	low risk of bias
Yetkin et al. [23], (2023)	Low (except for concerns about uncontrolled confounding)	low risk of bias	low risk of bias	low risk of bias	low risk of bias	low risk of bias	low risk of bias	low risk of bias
Gül et al. [24], (2024)	Low (except for concerns about uncontrolled confounding)	low risk of bias	low risk of bias	low risk of bias	low risk of bias	low risk of bias	low risk of bias	low risk of bias
Uzun et al. [9], (2022)	Low (except for concerns about uncontrolled confounding)	low risk of bias	low risk of bias	low risk of bias	low risk of bias	low risk of bias	low risk of bias	low risk of bias
Yasuda et al. [6], (2025)	Low (except for concerns about uncontrolled confounding)	low risk of bias	low risk of bias	low risk of bias	low risk of bias	low risk of bias	low risk of bias	low risk of bias
Huang et al. [11],(2025)	Low (except for concerns about uncontrolled confounding)	low risk of bias	low risk of bias	low risk of bias	low risk of bias	low risk of bias	low risk of bias	low risk of bias
Alis et al. [25], (2024)	Low (except for concerns about uncontrolled confounding)	low risk of bias	low risk of bias	low risk of bias	low risk of bias	low risk of bias	low risk of bias	low risk of bias
Emekli et al. [26], (2024)	Low (except for concerns about uncontrolled confounding)	low risk of bias	low risk of bias	low risk of bias	low risk of bias	low risk of bias	low risk of bias	low risk of bias
Celik et al. [27], (2021)	Low (except for concerns about uncontrolled confounding)	low risk of bias	low risk of bias	low risk of bias	low risk of bias	low risk of bias	low risk of bias	low risk of bias
Vagge et al. [10], (2022)	Low (except for concerns about uncontrolled confounding)	low risk of bias	low risk of bias	low risk of bias	low risk of bias	low risk of bias	low risk of bias	low risk of bias

## Discussion

The choroid is essential for sustaining oxygenation, thermal regulation, and the nutritional demands of the outer retina. Disruption of choroidal perfusion can compromise the retina and the foveal avascular zone, which is largely dependent on the choroid [33]. Research has shown that the choroid is susceptible to various systemic and ocular influences, including surgical interventions such as cataract and glaucoma surgery [34, 35]. With current innovations in imaging devices, it has become possible to examine the choroidal layers in detail. Enhanced depth imaging OCT and OCTA, have facilitated noninvasive and reproducible evaluation of the choroidal layers and hemodynamic alterations. These imaging modalities have also improved our ability to detect subtle vascular responses to surgical manipulation, even when clinical symptoms are absent, offering valuable insight into postoperative ocular physiology.

Cataract surgery has been shown to cause a significant increase in SFCT, typically emerging within the first postoperative week and persisting for several months [36]. These changes are generally attributed to postoperative inflammation and reduced intraocular pressure, leading to enhanced ocular perfusion and choroidal thickening. Similarly, glaucoma filtering procedures such as trabeculectomy produce transient choroidal thickening associated with intraocular pressure reduction and increased perfusion pressure, which usually resolves within a month [37, 38].

These well-characterized postoperative vascular changes in other ophthalmic surgeries provide an important context for interpreting the choroidal response after strabismus surgery and highlight why potential choroidal alterations remain a subject of clinical interest.

This meta-analysis sought to evaluate current research on the effects of strabismus surgery on choroidal circulation and its vascular dynamics. Our results found that strabismus surgery did not result in a statistically significant alteration in SFCT. Also, we did not find any statistically significant changes in the CVI, suggesting that strabismus surgery may have a minimal impact on choroidal vascular structure. Pooled analysis revealed no statistically significant change in foveal choriocapillaris VD and choriocapillaris flow. Considerable heterogeneity was observed in analyses of choriocapillaris vessel density. This variability likely reflects methodological and clinical differences across studies, including patient age, type and extent of surgery, imaging platforms, scan sizes, segmentation algorithms, and follow-up timing. Studies ranged from pediatric to adult populations, and age-related differences in baseline choroidal perfusion may influence postoperative vascular responses. In addition, OCTA devices differ in their definitions of the choriocapillaris slab and flow-detection algorithms, which may contribute to inconsistent measurements. These factors collectively limit direct comparability across studies and underscore the need for standardized OCTA protocols in future research. Despite sources of heterogeneity, meta-regression in the current analysis did not demonstrate a statistically significant effect for follow-up duration, type of surgery, or number of muscles operated on SFCT and choroidal vascularity parameters changes. Overall, this evidence suggests that in the general population and considering various surgical procedures, strabismus surgery did not systematically induce measurable changes in SFCT, CVI, and the foveal choriocapillaris VD and flow in the intermediate postoperative period (7-180 days).

Several mechanisms have been proposed to explain the postoperative changes in choroidal thickness and vascularity observed in the literature. As strabismus procedures frequently involve the severance of anterior ciliary arteries, they can theoretically compromise anterior segment perfusion and cause compensatory upregulation of long posterior ciliary artery flow. Studies have demonstrated this autoregulatory response as an increase in peak systolic velocity and resistive index on Doppler ultrasonography; however, because the choroidal vasculature rapidly compensates, no association with SFCT alteration has been observed [39, 40]. This rapid compensatory ability underscores the resilience of the posterior segment circulation and may explain why structural choroidal metrics remain stable despite measurable changes in upstream hemodynamics.

Surgical trauma and manipulation of perimuscular tissues can be another contributing factor and may prompt inflammation, increase vascular permeability, and induce transient tissue edema [4, 41]. Studies have reported early increases in choroidal thickness and vascular parameters during the acute inflammatory response, particularly in the deeper capillary plexus or choriocapillaris, with normalization at a later point in the study indicating resolution of the inflammatory state [42]. Such transient inflammatory effects may also interact with individual patient factors—such as age, axial length, or baseline vascular tone—yet these influences appear insufficient to produce lasting alterations in choroidal morphology.

Research suggests that fornix-based incisions tend to spare the perilimbal conjunctival-Tenon’s junction, preserve episcleral vascular plexuses, and minimize inflammation relative to limbal-based approaches [43]. Our subgroup analyses demonstrated a significant reduction in SFCT in procedures using fornix-based approaches. Seven studies had used the fornix-based approach for surgery, of which 4 studies operated on the inferior oblique muscle. This may suggest that the fornix-based technique itself may not reflect on the SFCT and that the decrease of SFCT is the consequence of the inferior oblique’s proximity to the macula and possible greater impact on macular and choroidal hemodynamics. However, our meta-analysis demonstrated only non-significant trends toward SFCT reduction after IO surgery, with the largest recent prospective case series also concluding that choroidal index and luminal area changes after inferior oblique myectomy are transient, returning to baseline by final follow-up [8, 42]. This finding may have practical relevance for surgeons choosing between surgical approaches, as it suggests that technique-related choroidal changes—when present—are self-limited and unlikely to influence long-term macular health.

A recent investigation by Uzun et al. evaluated the correlation between retrobulbar circulatory changes using color Doppler ultrasonography after strabismus surgery [41]. According to their data, the Peak systolic velocity of the posterior ciliary artery increased one week post-operatively and returned to similar pre-operative values after one month. The resistive index of the ophthalmic artery also increased postoperatively. Other retrobulbar flow parameters, which were studied, did not show any significant variations. Moreover, these changes did not correlate with choroidal thickness, highlighting a dissociation between vascular compensation and measurable morphologic changes in the choroid. This dissociation suggests that choroidal thickness alone may not fully capture the complexity of postoperative vascular adaptation, reinforcing the value of multimodal imaging when studying ocular perfusion changes.

Several studies using optical coherence tomography (OCT) and enhanced-depth imaging (EDI) have reported transient postoperative increases in choroidal thickness, typically peaking within one to two weeks and returning to baseline over subsequent months. Yasuda et al. showed that SFCT and choroidal blood flow, measured by laser speckle flowgraphy, were significantly elevated at one week and one month postoperatively, but these changes were no longer present at four months. In contrast, retinal vessel density initially decreased at one week but similarly returned to baseline, emphasizing the transient and potentially compensatory nature of these vascular responses [39]. These findings collectively reinforce the concept that choroidal and retinal microcirculation possess robust autoregulatory mechanisms capable of restoring physiologic equilibrium after surgical manipulation.

Taken together, while there are plausible acute mechanisms for choroidal thickness and vascular change after strabismus surgery, compensation is generally rapid, and any morphometric effects are transient. From a clinical perspective, this indicates that routine postoperative OCT or OCTA monitoring may not be necessary in uncomplicated cases, as persistent alterations in choroidal perfusion appear unlikely.

To the best of our knowledge, this study is the first meta-analysis investigating the quantitative changes in choroidal thickness and vascular components following strabismus surgery. However, several limitations exist in our study. Choroidal vascular indices were assessed in only a few studies, some with small sample sizes, which may have limited the ability to detect statistically significant effects. Another limitation arises from the countries of study, most of which were from Asia. As the thickness of some ocular layers varies with ethnicity, the included studies may only represent a group of ethnicities based on their country of origin. Also, unmeasured or unreported confounders in studies (e.g., smoking status, systemic vascular health, and diurnal variation in measurement) could contribute to unexplained heterogeneity. Differences in device calibration, operator experience, and use of single-point versus multipoint measurement protocols introduce potential systematic bias. Some studies did not report interrater or interrater reliability coefficients, and manual measurements remain at risk for operator subjectivity. Furthermore, we could not analyze the parameters based on follow-up periods, as study periods ranged from 7 days to 6 months postoperatively. Future multicenter studies with standardized imaging protocols, broader ethnic representation, and longer follow-up intervals will be essential for fully characterizing postoperative choroidal behavior and confirming the long-term vascular safety of strabismus surgery.

## Conclusion

This systematic review and meta-analysis demonstrates that strabismus surgery does not produce consistent or sustained alterations in SFCT, CVI, or choriocapillaris perfusion during the intermediate postoperative period. Although individual studies report transient early postoperative changes—likely reflecting short-lived inflammatory or autoregulatory responses—these effects generally resolve and do not result in measurable long-term modifications to choroidal structure or microvascular function. Importantly, all included studies were conducted in individuals without pre-existing retinal or choroidal pathology. Therefore, while the observed stability of choroidal parameters across diverse surgical techniques, age groups, and imaging modalities is reassuring for the general population, the current evidence does not allow conclusions regarding vascular safety in patients with underlying chorioretinal disease. Future prospective, standardized, multicenter studies—including higher-risk populations—are required to more fully characterize postoperative choroidal dynamics and define vascular safety in these groups.

## Supplementary Information

Below is the link to the electronic supplementary material.

Supplementary Material 1

## Data Availability

The datasets used during the current study are available from the corresponding author on reasonable request.

## Abbreviations

SFCT: Subfoveal choroidal thickness
CVI: Choroidal vascularity index
CC: Choriocapillaris
VD: Vessel density
OCT: Optical coherence tomography
OCTA: Optical coherence tomography angiography
TCA: Total choroidal area
LA: Luminal area
SA: Stromal area
PRISMA: Preferred Reporting Items for Systematic Reviews and Meta-Analyses
ROBINS-I: Risk Of Bias In Non-randomized Studies of Interventions
