# Supplementary material for: Effects of strabismus surgery on choroidal blood flow: a systematic review and meta-analysis
Source: Int J Retina Vitreous. 2026 Feb 23;12:52. doi: 10.1186/s40942-026-00818-1 (PMC13041447; doi:10.1186/s40942-026-00818-1)
Supplement: Supplementary file 1 — Supplementary Material 1 [file 40942_2026_818_MOESM1_ESM.docx]

Supplementary Table 1. Search strategy and the specific keywords used.

| **Query** | **Results** (25 May 2025) |
| --- | --- |
| **Pubmed** | |
| ((("optical coherence tomography"[Tiab] OR "optical coherence tomograph*"[Tiab] OR (Optical [Tiab] AND Coherence [Tiab] AND tomography [Tiab]) OR "OCT"[Tiab] OR "Tomography, Optical Coherence"[ MeSH Terms] OR "choroid"[Title/Abstract] "choroidal vasc*"[Title/Abstract] OR "Fundus Oculi"[Title/Abstract] OR "choroidal vascularity index"[Tiab] OR "choroidal blood flow"[Tiab] OR "CVI"[Tiab] OR OR ((("tomography, optical coherence"[MeSH Terms] OR ("tomography"[Tiab] AND "optical"[Tiab] AND "coherence"[Tiab]) OR "optical coherence tomography"[Tiab] OR ("optical"[Tiab] AND "coherence"[Tiab] AND "tomography"[Tiab])) AND ("angiography"[MeSH Terms] OR "angiography"[Tiab] OR "angiographies"[Tiab] OR "angiography s"[Tiab])) OR "OCTA"[Tiab] OR "OCT angio*"[Tiab] OR "OCT-angio*"[Tiab] OR "OCT-A"[Tiab] OR "SS-OCTA"[Tiab] OR "SD-OCTA"[Tiab] OR " Optical Coherence Tomography Angio*"[Tiab] OR ("OCT"[Tiab] AND "angiograph*"[Tiab]) OR "Angio-OCT"[Tiab] OR "Angio OCT"[Tiab] OR "oct angio*"[Tiab]))) and ((((((((((((((((strabism*[MeSH Terms]) OR (strabism*[Title/Abstract])) OR ((strabismus[Title/Abstract] AND surgery[Title/Abstract]))) OR (recess*[Title/Abstract])) OR (resect*[Title/Abstract])) | 345 |
| **Scopus** | |
| ( TITLE-ABS-KEY ( strabismus AND surg* ) ) AND ( TITLE-ABS-KEY ( choroidal AND vascula* ) OR TITLE-ABS-KEY ( optical AND coherence AND tomography ) OR TITLE-ABS-KEY ( "optical coherence tomography" ) OR TITLE-ABS-KEY ( "optical coherence tomograph*" ) OR TITLE-ABS-KEY ( "OCT" ) OR TITLE-ABS-KEY ( "Tomography, Optical Coherence" ) OR TITLE-ABS-KEY ( choroidal AND thickness ) OR TITLE-ABS-KEY ( choroidal AND circulation ) ) | 319 |
| **Web of Science** | |
| (TS=("optical coherence tomography" OR "OCT (optical coherence tomography)" OR "optical coherence tomography angiography" OR "optical coherence tomographic  angiography") AND TS=(" strabismus” OR “strabismus surgery”) | 446 |
| **Total records** | 1110 |
| **Total records without duplicates** | 646 |
